# Time of Day-Specific Changes in Metabolic Detoxification and Insecticide Tolerance in the House Fly, *Musca domestica* L.

**DOI:** 10.3389/fphys.2021.803682

**Published:** 2022-01-07

**Authors:** Chunmei You, Zelin Li, Yuanzhi Yin, Naretuya Na, Xiwu Gao

**Affiliations:** Department of Entomology, China Agricultural University, Beijing, China

**Keywords:** *Musca domestica*, beta-cypermethrin, insecticide resistance, diel rhythms, detoxification

## Abstract

Both insects and mammals all exhibit a daily fluctuation of susceptibility to chemicals at different times of the day. However, this phenomenon has not been further studied in the house fly (*Musca domestica* L.) and a better understanding of the house fly on chronobiology should be useful for controlling this widespread disease vector. Here we explored diel time-of-day variations in insecticide susceptibility, enzyme activities, and xenobiotic-metabolizing enzyme gene expressions. The house fly was most tolerant to beta-cypermethrin in the late photophase at Zeitgeber time (ZT) 8 and 12 [i.e., 8 and 12 h after light is present in the light-dark cycle (LD)]. The activities of cytochrome P450, GST, and CarE enzymes were determined in the house flies collected at various time, indicating that rhythms occur in P450 and CarE activities. Subsequently, we observed diel rhythmic expression levels of detoxifying genes, and *CYP6D1* and *Md*α*E7* displayed similar expression patterns with enzyme activities in LD conditions, respectively. No diel rhythm was observed for *CYP6D3* expression. These data demonstrated a diel rhythm of metabolic detoxification enzymes and insecticide susceptibility in *M*. *domestica*. In the future, the time-of-day insecticide efficacy could be considered into the management of the house fly.

## Introduction

Multiple factors, including physical, chemical, and physiological aspects, may influence insecticide toxicity (Pszczolkowski et al., 2004). As early as the 1960s, Beck (1963) found that the time of the day at which potassium cyanide was used could influence German cockroaches mortality. Since then, many studies have declared that circadian rhythms or chronotoxicity are associated with insecticide susceptibility in some insects, including *Drosophila melanogaster* (Beaver et al., 2012), mosquitoes (Yang et al., 2010; Rund et al., 2013), *Blattella germanica* (Lin et al., 2014), *Acheta domesticus* (Justyna et al., 2018), *Cimex lectularius* (Khalid et al., 2019), *Triatoma infestans* (Varela et al., 2019), and *Spodoptera litura* (Zhang et al., 2021).

House flies, *Musca domestica* L., a global public health pest which can transmit various pathogens, are primarily controlled by insecticides (Scott et al., 2000, 2013; Khamesipour et al., 2018). However, house flies have developed resistance to various kinds of insecticides all around the world, threatening the efficacy of chemical control strategies (Ma et al., 2017, 2020; Zhang et al., 2018, 2019, 2020; Freeman et al., 2019; You et al., 2020, 2021). Therefore, it is urgent to maintain insecticide efficiency for as long as we can. Synthetic pyrethroids targeting a voltage-sensitive sodium channel gene (*Vssc*) remain the most widely used insecticides against house flies due to their effectiveness and safety. Both target-site *Vssc* insensitivity and detoxification changes were two crucial pyrethroid resistance mechanisms reported in house flies (Liu et al., 2006; Zhang et al., 2007, 2010; Scott, 2017). Some studies showed the rhythmic susceptibility to DDT (dichlorodiphenyltrichloroethane), organophosphate, and pyrethrum aerosols in the house fly (Sullivan et al., 1970; Shipp and Otton, 1976). Rhythmic susceptibility to some insecticides was always associated with the changed expression of detoxifying enzymes especially cytochrome P450 (CYP) monooxygenase (P450s) for *D*. *melanogaster*, glutathione *S*-transferases (GST) for *Anopheles gambie*, and carboxylesterases (CarE) for *C*. *lectularius* (Hooven et al., 2009; Balmert et al., 2014; Khalid et al., 2019). Hooven et al. (2009) identified that significant daily variations of UGT and ECOD activities were associated with propoxur susceptibility in *D*. *melanogaster*. Time of day-specific changes in GST activities were involved in DDT and deltamethrin resistance in the malaria *A*. *gambie* (Balmert et al., 2014). Based on these studies, it was worth better understanding diel rhythms of detoxification mechanisms in insecticide toxicity against the house fly. *CYP6G4*, *CYP6D1*, and *CYP6D3* were constitutively overexpressed in the house fly populations resistant to pyrethroids (Gao et al., 2012). Additionally, quantitative and qualitative-changed *Md*α*E7* have been found to be associated with pyrethroids resistance in the house fly (Zhang et al., 2007, 2010). However, whether diel rhythms of these detoxification enzymes are involved in diel rhythmic insecticide susceptibility remains unknown in the house fly.

In this study, we hypothesized that *M*. *domestica* adults exhibited diel rhythms of insecticide susceptibility, in synchrony with their rhythmic behavior of being active to eat during the daytime and being inactive to rest throughout the night. Beta-cypermethrin, as a representative member of type II pyrethroids, has been extensively used for house fly control for many years. It is meaningful to explore diel rhythms of insecticide susceptibility for more rational application of insecticides. To understand whether the daily variations in beta-cypermethrin sensitivity were due to the physiological effects of detoxifying enzymes, we investigated the diurnal variations in beta-cypermethrin susceptibility, metabolic enzyme activities, and several detoxification-related gene expressions in *M*. *domestica* across 24 h under environmental light:dark (LD) conditions.

## Materials and Methods

### House Fly Rearing

A filed strain (CFD) of house fly was collected from a dump in Beijing, China, in 1998 and reared under standard laboratory conditions (25 ± 1°C, RH 60–70%, 16L:8D) in cages with enough food and water. The time of the day in this study was set by Zeitgeber time (ZT) 0, 4, 8, 12, 16, 20, 24, where ZT0 and ZT16 were, respectively defined as when the lights turn on and off in the 16:8 LD condition.

### Chemicals

Beta-cypermethrin (95.2%) was purchased from Tianjin Longdeng Chemical Co., Ltd. 7-ethoxycoumarin (7-EC), 7-hydroxycoumarin (7-HC), phenylmethylsulfonyl (PMSF), dithiothreitol (DTT), phenythiourea (PTU), α-naphthyl acetate (α-NA), β-naphthyl acetate (β-NA),1-chloro-2,4-dinitrobezene (CDNB), reduced glutathione (GSH), and fast blue RR salt and sodium dodecyl sulfate (SDS) were obtained from Sigma Chemical Co. NADPH was obtained from Solarbio Life Sciences Co., Ltd. The other chemicals were purchased from commercial suppliers.

### Insecticide Treatment

In the first study, a method utilizing a residual film glass tube (diameter 4.6 cm, height 7.6 cm, inner surface 140 cm^2^) was used to assess susceptibility. Beta-cypermethrin was firstly dissolved in acetone and then diluted to a series of appropriate concentrations resulting in 10–90% house fly mortality. A total of 1 mL of the insecticide solution was applied to the tubes and then allowed to evaporate for 30 min on a rolling machine. Fifteen 4-day-old flies were placed in each tube with three replications. Median lethal concentration (LC_50_) for beta-cypermethrin of the CFD strain was obtained 24 h after treatment.

In the second study, 3-day-old females were sorted into five equal populations, and then treated with the above LC_50_ with 12–15 flies at each time point (ZT0, 4, 8, 12, 16, 20, and 24). Mortality was assessed 1 h after treatment.

### Biochemical Assays

The house flies were collected every 4 h in a day at ZT0, ZT4, ZT8, ZT12, ZT16, ZT20, and ZT24 with three replications at each point in time, and the protein concentrations of these samples were measured as described by the Bradford method (Bradford, 1976).

#### P450 Activity

The deethylase activity of P450 monooxygenase enzyme was assayed as described before with some modifications by using the fluorogenic substrate 7-EC (Yu, 2014). The abdomens of house flies were collected and then homogenized in ice-cold phosphate buffer (PB) (0.1 M, pH 7.5) containing glycerol, protease inhibitors like ethylene diamine tetraacetic acid (EDTA), DTT, PTU, PMSF, and then centrifuged at 4°C, 10,800 rpm for 20 min. The supernatant was used as the enzyme source. The reaction mixture consisted of 80 μL of 7-EC (0.5 mM), 10 μL of NADPH (9.6 mM), and 50 μL of crude enzyme. The reactions were started by adding the enzyme. Fluorescence detection of the reaction product 7-HC was then measured with 390 excitation and 460 emission for every 30 s for 15 min using Flx800 (Biotek, Winooski, United States).

#### Carboxylesterases Activity

CarE activity was measured by the description of Zhang et al. (2020) with minor modifications with both α-NA and β-NA as substrates. The house fly abdomens were prepared and homogenized in ice-cold PB (0.04 M, pH 7.0). The crude enzyme source was collected as before (2.4.1.). For each reaction, 90 μL of PB containing 0.01 M α-NA/β-NA and 25 μL of diluted crude enzyme was incubated at 37°C for 15 min in 96-well plates. The reaction was then stopped immediately by adding 45 μL of fast blue RR salt solution. The plates were measured spectrophotometrically at 600 nm (α-NA) or 555 nm (β-NA) using a microplate reader (SPECTRA max PLUS384), respectively.

#### Glutathione *S*-Transferases Activity

The specific catalytic activity assay of GSTs toward CDNB was tested using the method of Oppenoorth et al. (1979) with modifications. The house fly abdomens were homogenized in ice-cold PB (0.1 M, pH 6.5). The crude enzyme source was collected as before (2.4.1.). For each reaction, 200 μL of PB containing 0.6 mM CDNB and 3 mM reduced glutathione (GSH), and 10 μL of diluted crude enzyme were added into 96-well plates. The dynamic detection of GSTs activity was then immediately read at 340 nm for every 15 s for 20 min on a microplate reader (SPECTRA max PLUS384).

### Quantitative Real-Time PCR

Total RNA collected from the CFD strain house flies (ZT0, 4, 8, 12, 16, 20, 24) using a Trizol kit (Invitrogen, Carlsbad, CA) was utilized for cDNA synthesis with the reverse transcription kit (Takara Biotechnology, Dalian, China). Each reaction for Quantitative Real-Time PCR (qRT-PCR) was conducted in 20 μL containing 1 μL of cDNA template, 10 μL of 2 × SYBR Premix Ex Taq (Takara), 0.4 μL of forward/reverse primer (10 mM), 0.4 μL of Rox II, and 7.8 μL of nuclease-free water on an Applied Biosystems 7500 Real-Time PCR system (Foster City, CA, United States) as previously described (Zhang et al., 2018). All the primers are listed in Table 1. Three technical replications were conducted in our study. Finally, the relative mRNA expression data of selected genes normalized with *GAPDH* were analyzed using the 2^–△△Ct^ method (Livak and Schmittgen, 2001).

**TABLE 1:** Sequences of primers in this study.

Gene	Accession number	Primer (5′–3′)	References
*CYP6G4*	NM_001286882 XM_005188667	Forward: CGGTTTGGTTGTACGCGATC	You et al., 2021
		Reverse: TTCCACTCGTAAACACCGGG	
*CYP6D1*	AF064794.1	Forward: TTTTTGAATCGCAAATGCAC Reverse: GGGGGAAATACTGTGGGTCT	Pan et al., 2018
*CYP6D3*	NM_001286881 XM_005184123	Forward: CCAAACGCCATTACACGCAA	You et al., 2021
		Reverse: TGCCCACAACTTGCTCTTGA	
*Md*α*E7*	AF133341	Forward: CGCTTCCTACAATTACGCTTC	Zhang et al., 2018
		Reverse: CATCGGCATGGCTTACACC	
*GAPDH*	XM_005176112	Forward: GGTCATCATCTCCGCTCCATC	Zhang et al., 2018
		Reverse: CAGTGGTGGCATGGACAGTGG	

### Data Analysis

All insecticide assays, biochemical assays, and gene expression analyses were determined by ANOVA tests with the GraphPad InStat 3.0 software (GraphPad Software, San Diego, CA, United States), and it was recognized to be statistically significant with a *P*-value < 0.05.

## Results

### Rhythmic Susceptibility to Beta-Cypermethrin

**FIGURE 1:**
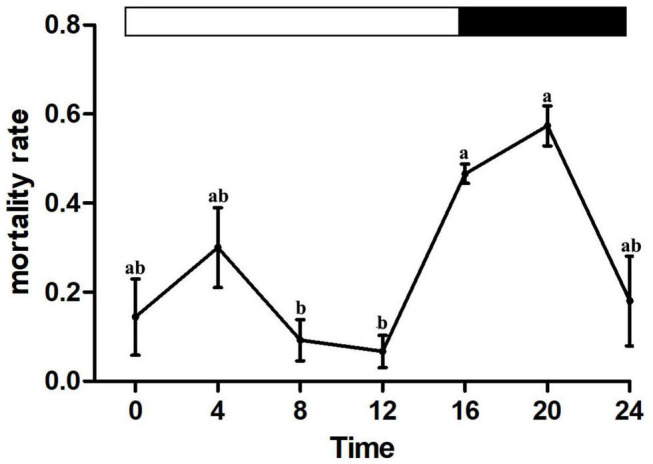
Diel rhythms of house fly susceptibility to beta-cypermethrin. Error bars display SEM. Letters above the error bars devote significant differences (one-way ANOVA and Tukey’s test, *P* < 0.05).

All individuals who were unconscious at 1 h after treatment did not recover consciousness after 24 h. Differences in house fly mortality of those exposed to LC_50_ 1.45 μg/cm^2^ beta-cypermethrin at different times of day indicate that the house flies possessed rhythmic susceptibility to this insecticide (Figure 1). The house flies showed lower susceptibility in the photophase than in the scotophase with the lowest mortality at ZT8 and ZT12 (Figure 1). These results implied that *M*. *domestica* increased tolerance to beta-cypermethrin during their active state in the light phase of the photoperiod.

### Daily Rhythms in Enzymatic Activity

**FIGURE 2:**
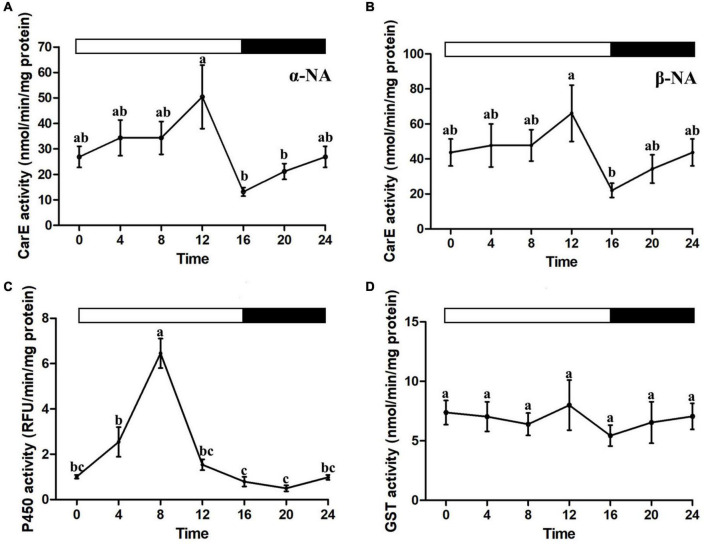
Diel rhythms of the house fly detoxification enzyme activities. **(A,B)** Carboxylesterase enzyme activity. **(C)** P450 enzyme activity. **(D)** Glutathione S-transferase enzyme activity. Error bars display SEM. Letters above the error bars devote significant differences (one-way ANOVA and Tukey’s test, *P* < 0.05).

Biochemical assays for cytochrome P450, CarE, and GSTs activities were performed in the house flies collected every 4 h over a diel cycle (one-way ANOVA, *P* < 0.05, Figure 2). Time of day-specific changes in P450 and CarE activities were observed. CarE activities possessed similar patterns between α-NA and β-NA substrates, with peaks at ZT12 and lows at ZT16 (Figures 2A,B). Cytochrome P450 enzyme activity continuously increased in the early photophase and reached the highest recorded level at ZT8 (Figure 2C). No daily changes in GST activity were found (Figure 2D).

### Rhythmic Gene Expression

**FIGURE 3:**
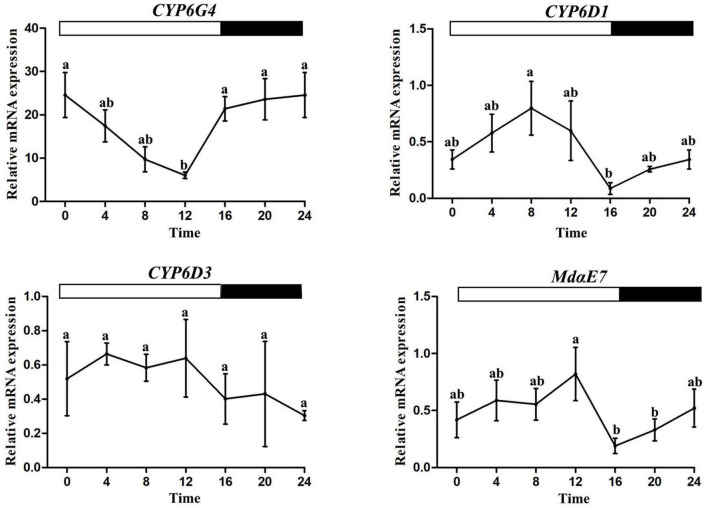
Rhythmic expression patterns of metabolic detoxification genes. Error bars display SEM. Letters above the error bars devote significant differences (one-way ANOVA and Tukey’s test, *P* < 0.05).

qRT-PCR was used to determine the rhythmic expression of four selected detoxification genes (*CYP6G4, CYP6D1, CYP6D3, Md*α*E7*) which were associated with pyrethroid resistance in the house fly. One-way ANOVAs showed significant time-of-day differences in mRNA expression levels of *CYP6D1, CYP6G4*, and *Md*α*E7* except *CYP6D3*. *CYP6D1* and *Md*α*E7* expression displayed similar daily fluctuations with a trough at ZT16, and a peak at ZT8 and ZT12 separately (one-way ANOVA, *P* < 0.05, Figure 3). However, *CYP6G4* expression decreased during the photophase, reaching the bottom at ZT12 (one-way ANOVA, *P* < 0.05, Figure 3).

## Dicussion

In this study, we found that house flies displayed diel rhythms in beta-cypermethrin susceptibility, peaking at ZT16 and ZT20, and the house flies were consistently insusceptible throughout the day in the 16:8 LD cycle diel conditions which was consistent with natural conditions. The research of Shipp and Otton (1976) suggested that the tolerance of house flies to different insecticides was diel-rhythmic and the most tolerant was observed at 1 h from the onset of light, indicating that the “time of greatest susceptibility occurs at about the time of onset of increased activity at dawn.” However, some other studies suggested that the time of the greatest susceptibility was dependent on the species and the insecticide action mode. Some insects displayed peak resistance to insecticides in the photophase of the light/dark condition, for example, *D*. *melanogaster* (ZT4 for propoxur and fipronil in 12:12 LD cycle) (Hooven et al., 2009), *Aedes aegypti* (ZT9 for permethrin in 12:12 LD cycle) (Yang et al., 2010), and *A*. *gambiae* (ZT10.6 for DDT in 12:12 LD cycle, and ZT0.6 and ZT10.7 for deltamethrin in 12:12 LD cycle) (Balmert et al., 2014). On the opposite side, some insects displayed peak survival in the dark-phase of the photoperiod, for instance, *D*. *melanogaster* (ZT20 for malathion in 12:12 LD cycle) (Hooven et al., 2009), *Blattella germanica* (ZT21 for permethrin in 12:12 LD cycle) (Lin et al., 2014), and *D*. *suzukii* (ZT0 for malathion in 14:10 LD cycle) (Hamby et al., 2013).

The detoxifying pathways were basically responsible for metabolizing toxic chemicals in most biological systems. The activities of P450, GST, and CarE enzymes were investigated in our study for determining whether there was a relationship between the diel rhythms in beta-cypermethrin susceptibility and physiological results of detoxification enzymes. The patterns of enzyme activities reached the peak at ZT8 for P450 and ZT12 for CarE separately, which were in accordance with the diel rhythms examined in the beta-cypermethrin assay, suggesting that P450 and CarE probably mediate the daily fluctuations of beta-cypermethrin susceptibility in the house fly.

It has reported that the rhythmic expression of detoxification enzyme genes (*CYP6M2*, *CYP6P3*, *CYP6Z1*, *CYP6P3*, and *GSTD7*) were implicated in rhythmic insecticidal detoxification in mosquitoes (Rund et al., 2013; Balmert et al., 2014). *BgGSTD1* was responsible for the diel regulation of permethrin susceptibility in the German cockroach (Lin et al., 2014). Based on these findings, we investigated the expression patterns of previously reported important P450 (*CYP6G4*, *CYP6D1*, *CYP6D3*) and CarE (*Md*α*E7*) genes. The data suggested a potential relationship between the diel rhythms of these genes and insecticide susceptibility to beta-cypermethrin. For *Md*α*E7*, the mRNA expression pattern was similar to the result of the beta-cypermethrin toxicity assay, peaking at ZT12. *CYP6D1* was rhythmically expressed in the 16:8 light/dark condition with the highest expression level at ZT8. The genes involved in metabolic and detoxification pathways were expressed at different times throughout the day rather than clustering at a special time. Our data suggested that the gene expression patterns of *CYP6D1* and *Md*α*E7* were consistent with the daily fluctuations of P450 and CarE enzyme activities, displaying the highest point at ZT8 and ZT12 separately in the LD condition. However, *CYP6D3*, implicated in detoxification of pyrethroids, did not have rhythmic mRNA expression levels. *CYP6G4* was probably under a different diel regulation without association with insecticidal detoxification. In a similar study, the relative mRNA expression level of *CE3959* was not consistent with the rhythmic expression of esterase activity in *C*. *lectularius* resistant to deltamethrin (Khalid et al., 2019). Therefore, it was not surprising that *CYP6D3* and *CYP6G4* do not correspond to the observations of the beta-cypermethrin assay and P450s activity, suggesting that some other candidate cytochrome P450 genes were probably associated with the tested P450 activity.

Circadian clocks have been found in many organisms for their adaptations to periodic alternations, and to orchestrate metabolic and behavioral activities (Bazalova and Dolezel, 2017). Numerous genes, including rhythmic detoxifying genes, were in the regulation of concerted action of endogenous circadian clock genes (Lin et al., 2002; Beaver et al., 2010; Rund et al., 2011, 2013). Yang et al. (2010) demonstrated that the daily fluctuation of permethrin resistance in *Aedes aegypti* was correlated with the expression of *CYP9M9* and the clock gene *period*. There was a link between the rhythmic activity of glutathione levels and the clock genes (*cycle* and *period*) in *D*. *melanogaster* (Williams and Sehgal, 2001; Beaver et al., 2012). There were some similarities and differences of the circadian rhythm gene regulation between the house fly and other dipteran insects (Codd et al., 2007; Sandrelli et al., 2010). Feeding, light, and temperature were crucial cues driving the circadian system. The temperature targeting *period* gene possessed an impact on the locomotor activity in the house fly (Bazalova and Dolezel, 2017). Arylalkylamine n-acetyltransferase (aanat) via melatonin/mt2-like receptor can function as a connection between the circadian rhythms and locomotor activity in the brain of the American cockroach (Kamruzzaman et al., 2021). The house flies were inactive in the darkness phase with increased beta-cypermethrin susceptibility and reduced detoxifying enzyme activities, which can be explained by their lower possibilities of exposure to exogenous chemicals. Diel rhythmic expression levels of detoxifying genes consistent with daytime activity were observed for *CYP6D1* and *Md*α*E7*. The strong circadian control of insect activities was associated with cytochrome P450s in that CYPs require heme as a prosthetic group and heme availability was strongly circadian (Zmrzljak and Rozman, 2012). It has been reported that circadian elements can activate the transcription of detoxification genes by directly binding to responding E-boxes of gene promoter regions (Zhang et al., 2021). However, whether the insecticide-metabolizing genes *CYP6D1* and *Md*α*E7* are analogously E-box-regulated and the link to the circadian clock need further research. Our study started with the rhythms of beta-cypermethrin resistance and then found a relationship between detoxification metabolism and resistance level in *M*. *domestica* under environmental light:dark conditions. The house fly was most tolerant to beta-cypermethrin in the late photophase at ZT8 and ZT12. Rhythms also occurred in P450 and CarE activities. Both *CYP6D1* and *Md*α*E7* genes displayed similar expression patterns with enzyme activities in the given LD condition, whereas no diel rhythm was observed for *CYP6D3* and *CYP6G4* expression which may be a response to non-circadian physiological metabolism. This proposed that the association with insecticide resistance and daytime xenobiotic detoxification contributes to intensive daytime feeding or other activities in *M*. *domestica* which can enable it to evade the risks of insecticides. Given the geographically widespread health damage of this unsanitary pest, these results have advanced the understanding of the molecular basis to adapt to adverse environmental conditions and promises to contribute to more effective control of this pest.

## Data Availability Statement

The original contributions presented in the study are included in the article/supplementary material, further inquiries can be directed to the corresponding author/s.

## Author Contributions

CY conceived the study, conducted the experiments, and drafted the manuscript. ZL and YY conducted the experiments and analyzed the data. NN revised the article. XG conceived the study and approved the final manuscript.

## Conflict of Interest

The authors declare that the research was conducted in the absence of any commercial or financial relationships that could be construed as a potential conflict of interest.

## Publisher’s Note

All claims expressed in this article are solely those of the authors and do not necessarily represent those of their affiliated organizations, or those of the publisher, the editors and the reviewers. Any product that may be evaluated in this article, or claim that may be made by its manufacturer, is not guaranteed or endorsed by the publisher.

## References

[B1] Balmert N. J. Rund S. S. C. Ghazi J. P. Zhou P. Duffield G. E. (2014). Time-of-day specific changes in metabolic detoxification and insecticide resistance in the malaria mosquito *Anopheles gambiae*. *J. Insect Physiol.* 64 30–39. 10.1016/j.jinsphys.2014.02.013 24631684

[B2] Bazalova O. Dolezel D. (2017). Daily activity of the housefly, *Musca Domestica*, is influenced by temperature independent of 3′ utr period gene splicing. *G3* 7 2637–2649. 10.1534/g3.117.042374 28620087 PMC5555469

[B3] Beaver L. M. Hooven L. A. Butcher S. M. Krishnan N. Sherman K. A. Chow E. S. (2010). Circadian clock regulates response to pesticides in *Drosophila* via conserved *Pdp1* pathway. *Toxicol. Sci.* 115 513–520. 10.1093/toxsci/kfq083 20348229 PMC2871760

[B4] Beaver L. M. Klichko V. I. Chow E. S. Kotwica-Rolinska J. Williamson M. Orr W. C. (2012). Circadian regulation of glutathione levels and biosynthesis in *Drosophila melanogaster*. *PLoS One* 7:e50454. 10.1371/journal.pone.0050454 23226288 PMC3511579

[B5] Beck S. D. (1963). Physiology and ecology of photoperiodism. *Bull. Entomol. Soc. Am.* 9 8–16. 10.1093/besa/9.1.8

[B6] Bradford M. M. (1976). A rapid and sensitive method for the quantification of microgram quantities of protein utilizing the principle of protein dye binding. *Anal. Biochem.* 72 248–254. 10.1006/abio.1976.9999 942051

[B7] Codd V. Dolezel D. Stehlik J. Piccin A. Garner K. J. Racey S. N. (2007). Circadian rhythm gene regulation in the house fly *Musca domestica*. *Genetics* 177 1539–1551. 17947418 10.1534/genetics.107.079160 PMC2147977

[B8] Freeman J. C. Ross D. H. Scott J. G. (2019). Insecticide resistance monitoring of house fly populations from the United States. *Pestic. Biochem. Physiol.* 158 61–68. 10.1016/j.pestbp.2019.04.006 31378362

[B9] Gao Q. Li M. Sheng C. F. Scott J. G. Qiu X. H. (2012). Multiple cytochrome P450s overexpressed in pyrethroid resistant house flies (*Musca domestica*). *Pestic. Biochem. Physiol.* 104 252–260. 10.1016/j.pestbp.2012.09.006

[B10] Hamby K. A. Kwok R. S. Zalom F. G. Chiu J. C. (2013). Integrating circadian activity and gene expression profiles to predict chronotoxicity of *Drosophila suzukii* response to insecticides. *PLoS One* 8:e68472. 10.1371/journal.pone.0068472 23861907 PMC3702611

[B11] Hooven L. A. Sherman K. A. Butcher S. Giebultowicz J. M. (2009). Does the clock make the poison? Circadian variation in response to pesticides. *PLoS One.* 4:e6469. 10.1371/journal.pone.0006469 19649249 PMC2714471

[B12] Justyna M. Bartosz P. Gabriela M. Lech Z. Sonia M. (2018). Pyrethroid residue dynamics in insects depends on the circadian clock. *J. Environ. Sci. Health B.* 53 441–446. 10.1080/03601234.2018.1439336 29485346

[B13] Kamruzzaman A. S. M. Hiragaki S. Watari Y. Natsukawa T. Yasuhara A. Ichihara N. (2021). Clock-controlled arylalkylamine n-acetyltransferase (aanat) regulates circadian rhythms of locomotor activity in the american cockroach, periplaneta americana, via melatonin/mt2-like receptor. *J. Pineal Res.* 71:e12751. 10.1111/jpi.12751 34091948

[B14] Khalid M. F. Lee C. Y. Doggett S. L. Singham G. V. (2019). Circadian rhythms in insecticide susceptibility, metabolic enzyme activity, and gene expression in *Cimex lectularius* (*Hemiptera: Cimicidae*). *PLoS One* 14:e0218343. 10.1371/journal.pone.0218343 31206537 PMC6576784

[B15] Khamesipour F. Lankarani K. B. Honarvar B. Tebit K. E. (2018). A systematic review of human pathogens carried by the house fly (*Musca domestica L.*). *BMC Public Health* 18 1049–1063. 10.1186/s12889-018-5934-3 30134910 PMC6104014

[B16] Lin Y. H. Lee C. M. Huang J. H. Lee H. J. (2014). Circadian regulation of permethrin susceptibility by glutathione Stransferase (*BgGSTD1*) in the German cockroach (*Blattella germanica*). *J. Insect Physiol.* 65 45–50. 10.1016/j.jinsphys.2014.05.001 24819204

[B17] Lin Y. Han M. Shimada B. Wang L. Gibler T. M. Amarakone A. (2002). Influence of the period dependent circadian clock on diurnal, circadian, and aperiodic gene expression in *Drosophila melanogaster*. *Proc. Natl. Acad. Sci. U.S.A.* 99 9562–9567. 10.1073/pnas.132269699 12089325 PMC123180

[B18] Liu N. N. Zhu F. Xu Q. Pridged J. W. Gao X. W. (2006). Behavioral change, physiological modification, and metabolic detoxification: mechanisms of insecticide resistance. *Acta Entomol. Sinica* 49 671–679.

[B19] Livak K. J. Schmittgen T. D. (2001). Analysis of relative gene expression data using real time quantitative PCR and the 2^–ΔΔCT^ method. *Methods* 25 402–408. 10.1006/meth.2001.1262 11846609

[B20] Ma Z. Li J. Zhang Y. Shan C. Gao X. W. (2017). Inheritance mode and mechanisms of resistance to imidacloprid in the house fly *Musca domestica* (*Diptera: Muscidae*) from China. *PLoS One* 12:e0189343. 10.1371/journal.pone.0189343 29228021 PMC5724887

[B21] Ma Z. Zhang Y. You C. M. Zeng X. P. Gao X. W. (2020). The role of g protein-coupled receptor-related genes in cytochrome p450-mediated resistance of the house fly, *Musca domestica (Diptera: Muscidae*), to imidacloprid. *Insect Mol. Biol.* 29 92–103. 10.1111/imb.12615 31456272

[B22] Oppenoorth F. J. Van der Pas L. J. T. Houx N. W. H. (1979). Glutathione *S*-transferase and hydrolytic activity in a tetrachlorvinphos-resistant strain of house fly and their influence on resistance. *Pestic. Biochem. Physiol.* 111 176–178. 10.1016/0048-3575(79)90057-9

[B23] Pan J. Yang C. Liu Y. Gao Q. Li M. Qiu X. (2018). Novel cytochrome P450 (CYP6D1) and voltage sensitive sodium channel (Vssc) alleles of the house fly (*Musca domestica*) and their roles in pyrethroid resistance. *Pest Manag. Sci.* 7 978–986. 10.1002/ps.4798 29155487

[B24] Pszczolkowski M. A. Dobrowolski M. Spencer C. (2004). When did you last test your insects? The forgotten importance of chronotoxicology. *Am. Entomol.* 50 72–74.

[B25] Rund S. S. C. Gentile J. E. Duffield G. E. (2013). Extensive circadian and light regulation of the transcriptome in the malaria mosquito *Anopheles Gambiae*. *BMC Genomics* 14:1–19. 10.1186/1471-2164-14-218 23552056 PMC3642039

[B26] Rund S. S. C. Hou T. Y. Ward S. M. Collins F. H. Duffield G. E. (2011). Genome-wide profiling of diel and circadian gene expression in the malaria vector *Anopheles gambiae*. *Proc. Natl. Acad. Sci. U.S.A.* 108 421–430. 10.1073/pnas.1100584108 21715657 PMC3156198

[B27] Sandrelli F. Costa R. Kyriacou C. P. Rosato E. (2010). Comparative analysis of circadian clock genes in insects. *Insect Mol. Biol.* 17 447–463. 10.1111/j.1365-2583.2008.00832.x 18828836

[B28] Scott J. G. (2017). Evolution of resistance to pyrethroid insecticides in *Musca domestica*. *Pest Manag. Sci.* 73 716–722. 10.1002/ps.4328 27241012

[B29] Scott J. G. Alefantis T. G. Kaufman P. E. Rutz D. A. (2000). Insecticide resistance in house flies from caged-layer poultry facilities. *Pest Manag. Sci.* 56 147–153. 10.1002/(sici)1526-4998(200002)56:2<147::aid-ps106>3.0.co;2-7

[B30] Scott J. G. Leichter C. A. Rinkevihc F. D. Harris S. A. Su C. Aberegg L. C. (2013). Insecticide resistance in house flies from the United States: resistance levels and frequency of pyrethroid resistance alleles. *Pestic. Biochem. Physiol.* 107 377–384. 10.1016/j.pestbp.2013.10.006 24267700

[B31] Shipp E. Otton J. (1976). Circadian rhythms of sensitivity to insecticides in *Musca domestica* (*Diptera. Muscidae*). *Entomol. Exp. Appl.* 19 163–171. 10.1111/j.1570-7458.1976.tb02593.x

[B32] Sullivan W. N. Cawley B. Hayes D. K. (1970). Circadian rhythm in susceptibility of house flies and madeira cockroaches to pyrethrum. *J. Econ. Entomol.* 63 159–163. 10.1093/jee/63.1.159 5435525

[B33] Varela G. M. Stroppa M. M. García B. A. (2019). Daily variations in the expression of genes related to insecticide resistance in the chagas disease vector *Triatoma infestans* (*Hemiptera: Reduviidae*). *Am. J. Trop. Med. Hyg.* 100 1482–1485. 10.4269/ajtmh.19-0155 30994101 PMC6553907

[B34] Williams J. A. Sehgal A. (2001). Molecular components of the circadian system in *Drosophila*. *Annu. Rev. Physiol.* 63 729–755. 11181974 10.1146/annurev.physiol.63.1.729

[B35] Yang Y. Y. Liu Y. Teng H. J. Sauman I. Sehnal F. Lee H. J. (2010). Circadian control of permethrin-resistance in the mosquito *Aedes aegypti*. *J. Insect Physiol.* 56 1219–1223. 20361972 10.1016/j.jinsphys.2010.03.028

[B36] You C. M. Shan C. Ma Z. Zhang Y. Zhao R. Gao X. W. (2021). The overexpression and variant of CYP6G4 associated with propoxur resistance in the housefly, *Musca domestica L*. *Pest Manag. Sci*. 77 4321–4330. 10.1002/ps.6461 33942965

[B37] You C. M. Shan C. Xin J. J. Li J. Ma Z. Zhang Y. (2020). Propoxur resistance associated with insensitivity of acetylcholinesterase (ache) in the housefly, *Musca domestica (Diptera: Muscidae*). *Sci. Rep.* 10:8400. 10.1038/s41598-020-65242-3 32439946 PMC7242383

[B38] Yu W. T. (2014). *Mechanisms Of Field-Evolved Resistance To Fenvalerate And Resistance Risk Assessment Of Insecticides In Helicoverpa Armigera*. Master degree, Nanjing: Nanjing Agricultural University.

[B39] Zhang J. Li S. Li W. Chen Z. Mita K. (2021). Circadian regulation of night feeding and daytime detoxification in a formidable asian pest *Spodoptera litura*. *Commun. Biol.* 4:286. 10.1038/s42003-021-01816-9 33674721 PMC7935888

[B40] Zhang L. Gao X. W. Liang P. (2007). Beta-cypermethrin resistance associated with high carboxylesterase activities in a strain of housefly. *Musca domestica (Diptera: Muscidae*). *Pestic. Biochem. Physiol*. 89 65–72.

[B41] Zhang L. Shi J. Shi X. Liang P. Gao J. Gao X. W. (2010). Quantitative and qualitative changes of the carboxylesterase associated with beta-cypermethrin resistance in the house fly, *Musca domestica* (*Diptera: Muscidae*). *Comp. Biochem. Phys. B.* 156 6–11. 10.1016/j.cbpb.2010.01.011 20117228

[B42] Zhang Y. Guo M. C. Ma Z. You C. M. Shi X. Y. Gao X. W. (2020). Esterase-mediated spinosad resistance in house flies *Musca domestica (Diptera: Muscidae*). *Ecotoxicology* 29 35–44. 10.1007/s10646-019-02125-y 31749037

[B43] Zhang Y. Li J. Ma Z. Shan C. Gao X. W. (2018). Multiple mutations and overexpression of the *MdαE7* carboxylesterase gene associated with male-linked malathion resistance in house fly, *Musca domestica (Diptera: Muscidae*). *Sci. Rep.* 8:224. 10.1038/s41598-017-17325-x 29317643 PMC5760516

[B44] Zhang Y. Wang Y. Ma Z. Zhai D. Gao X. W. Shi X. Y. (2019). Cytochrome P450 monooxygenases-mediated sex-differential spinosad resistance in house flies *Musca domestica (Diptera: Muscidae*). *Pestic. Biochem. Physiol.* 157 178–185. 10.1016/j.pestbp.2019.03.024 31153466

[B45] Zmrzljak U. P. Rozman D. (2012). Circadian regulation of the hepatic endobiotic and xenobiotic detoxification pathways: the time matters. *Chem. Res. Toxicol.* 25 811–824. 10.1021/tx200538r 22303888

